# ExPRESS miniature glaucoma shunt for intractable secondary glaucoma in superior vena cava syndrome - a case report

**DOI:** 10.1186/s12886-016-0301-6

**Published:** 2016-07-26

**Authors:** Yi-Ju Ho, Chi-Hsiao Yeh, Chi-Chun Lai, Jerry Chien-Chieh Huang, Lan-Hsin Chuang

**Affiliations:** 1 Department of Ophthalmology, Chang Gung Memorial Hospital, Linkou. No. 5, Fuxing St, Guishan Dist Taoyuan City, 333 Taiwan, Republic of China; 2 Department of Ophthalmology, Chang Gung Memorial Hospital, Keelung. 222 Mai-Chin Rd, Keelung, 204 Taiwan, Republic of China; 3 Department of Thoracic and Cardiovascular Surgery, Chang Gung Memorial Hospital, Keelung. 222 Mai-Chin Rd, Keelung, 204 Taiwan, Republic of China; 4 College of Medicine, Chang Gung University, 259, Wenhua 1st Rd, Guishan Dist Taoyuan City, 33302 Taiwan, Republic of China

**Keywords:** Secondary glaucoma, Superior vena cava syndrome, Trabeculectomy, Express

## Abstract

**Background:**

The aim of this study was to clarify the pathogenic mechanism and to evaluate an intervention for intractable secondary glaucoma in superior vena cava (SVC) syndrome.

**Case presentation:**

A 66-year-old female with underlying hypertension, diabetes mellitus, ischaemic heart disease and end-stage renal disease complained of bilateral puffy eyelids for 3 months. Over three years, the patient experienced a progressive, marked face and neck swelling, which was accompanied by dyspnoea and nocturnal coughing. The patient has been under haemodialysis for the past 5 years; there were several occurrences of vascular access re-establishment for susceptibility to vascular thrombosis, and she was also diagnosed with SVC syndrome 2 years after haemodialysis. The patient’s best-corrected visual acuity (BCVA) was 20/60 in the right eye and 20/400 in the left eye. Pneumatic tonometry revealed a gradual increase in the intraocular pressure (IOP), even with the use of three types of anti-glaucoma agents. The ratio was 0.7 and bilaterally symmetric; optical coherence tomography indicated a thinning of the superior and inferior retina nerve fibre layers, and standard automated perimetry showed partial to generalized depression in both eyes.

Filtering surgery for the left eye was performed, but postoperatively, the IOP increased gradually over three months. The subsequent placement of the ExPRESS miniature glaucoma device p200 effectively lowered the IOP. Postoperatively, the IOP of the left eye remained under 20 mmHg without a further decrease in visual acuity, while the right eye, which was controlled with only medication, had an IOP of greater than 30 mmHg. Because this patient refused cardiovascular intervention, conventional trabeculectomy was used to redirect the aqueous humour to the subconjunctival space to form a bleb, but failed. Fortunately, the subsequent ExPRESS implant effectively facilitated aqueous outflow through the intrascleral space, resulting in the maintenance of a normal IOP at 6 months, postoperatively.

**Conclusion:**

Sustained high IOP may occur after conventional filtration surgery for secondary glaucoma in SVC syndrome. To facilitate aqueous outflow, an ExPRESS glaucoma implant can be used to effectively control the IOP.

## Background

Even though episcleral vein occlusion has been used to increase the intraocular pressure (IOP) in an animal model [1, 2], there are only a few in vivo studies on the course and management of such cases. Causes of obstruction of venous drainage that affect the eyes include episcleral vein obstruction, orbital disease, cavernous sinus thrombosis, jugular vein occlusion and superior vena cava obstruction. In particular, for patients under chronic haemodialysis, it is not uncommon that superior vena cava (SVC) syndrome may develop due to venous stenosis [3, 4].

In SVC syndrome cases, there is an obstruction of blood flow through the superior vena cava; and dyspnoea, chest pain, face edema, neck and eyelid swelling and ocular hypertension can occur because of the impaired venous drainage of body fluid. Nevertheless, Yu et al. previously proposed a classification for SVC syndrome; according to this report, visual symptoms are rare, and if visual disturbance by ocular oedema is present, the disease is graded as being in a moderate stage [5].

The drainage of the aqueous humour into the venous system improves the balance of the intraocular pressure and visual function. A blockade of the venous drainage is one of the rare causes of glaucoma with elevated episcleral pressure in SVC syndrome and might not be noticed due to its subtle ocular manifestation, especially before and after venous drainage impairment has been resolved. Thus far, a few case reports on ocular manifestations in SVC syndrome exist, but few studies have reported treatment for the eyes with elevated episcleral vein pressure.

Therefore, the purpose of this retrospective study was to clarify the underlying pathogenic mechanism and to evaluate the effective interventions of secondary glaucoma in SVC syndrome. In addition, we will establish the efficacy and safety of surgical intervention for secondary glaucoma in SVC syndrome.

## Case presentation

**Fig. 1:**
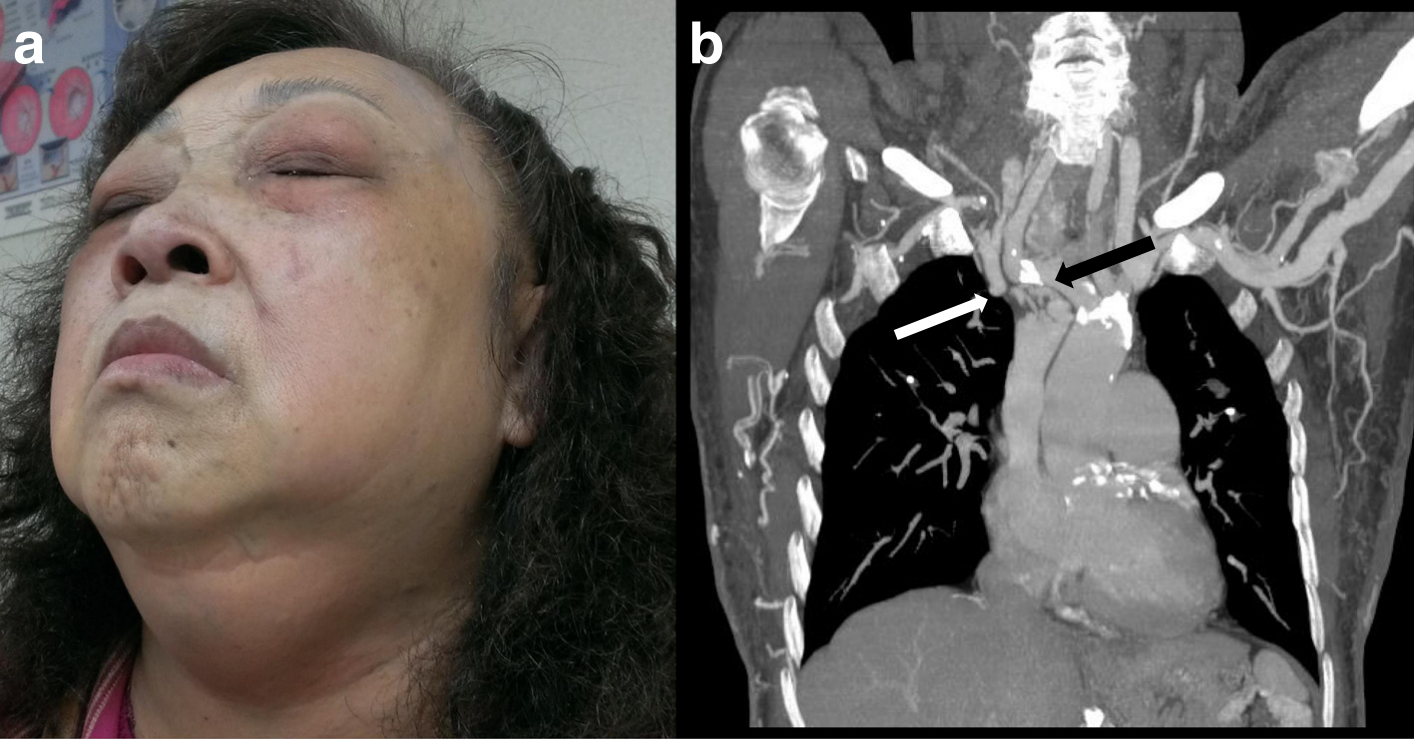
SVC syndrome occurred several times after repeated implantation of tunnelled double-lumen catheters for haemodialysis. **a** Gradual development of SVC syndrome with classic symptoms, including obvious face, neck, and upper trunk swelling. **b** A reconstructed coronal section of the contrast enhanced computed tomography angiogram revealed a fibrotic SVC. The occlusion (white arrow) of the right internal jugular vein at the junction with SVC and accompanying collateral blood-flow network (black arrow) indicated chronic SVC obstruction. (SVC = superior vena cava)

A 66-year-old woman presented to our outpatient services in 2011 with the complaint of progressively puffy eyelids on both eyes in the most recent 3 months. Her past medical history showed hypertension, type 2 diabetes mellitus, ischaemic heart disease, and end-stage renal disease. Additionally, she had been undergoing haemodialysis three times a week since 2009 (3 years ago). With the history of chronic haemodialysis, in 2011, a computed tomography angiography (CTA) revealed an occlusion of the right subclavian vein at the junction with the superior vena cava (SVC), and there was a subtotal occlusion at the SVC orifice in the arteriography (Fig. 1b). Progressively, her appearance changed, with marked oedema of the face and neck, but without any obvious lymphadenopathy or palpable mass on the neck (Fig. 1a). Additionally, she experienced dyspnoea and intermittent nocturnal coughing. The patient received cataract surgery for both eyes 10 years ago. There was no use of corticosteroids in her medical records and no positive family history of glaucoma.

A general inspection revealed the orthotropic primary ocular position without proptosis. The eyelids were swollen without tenderness or local heat, and initially, the intraocular pressure was normal. There was moderate congestion of the conjunctiva in both eyes; the corneas were clear, and the anterior chambers were deep and quiet. In 2013, two years after SVC syndrome was diagnosed, pneumatic tonometry revealed bilateral high IOP (22 mmHg OD, 23 mmHg OS) compared to a prior visit. Then, the IOP increased progressively to 30 mmHg, even with the combined use of brimonidine, dorzolamide and latanoprost, which was aimed at controlling IOP. At that time, the patient’s best-corrected visual acuity (BCVA) was 20/60 in the right eye and 20/400 in the left eye. Gonioscopy demonstrated wide-open angles in both eyes without peripheral anterior synechiae or neovascularization of the angle. Upon fundus examination, the cup-to-disk ratio was found to have increased to 0.7 in both eyes after one and half years of treatment with an anti-glaucoma agent. Optical coherence tomography showed the superior and inferior thinning of the retinal nerve fibre layer (RNFL) in both eyes, which was worse on the left side. Specifically, the patient had an average RNFL of 65.57 μm in the right eye and 47.51 μm in the left eye. Standard automated perimetry showed a three-fourths quadrant depression in the right eye and more generalized depression in the left eye; the mean deviations were -17.57 and -27.07, respectively.

**Fig. 2:**
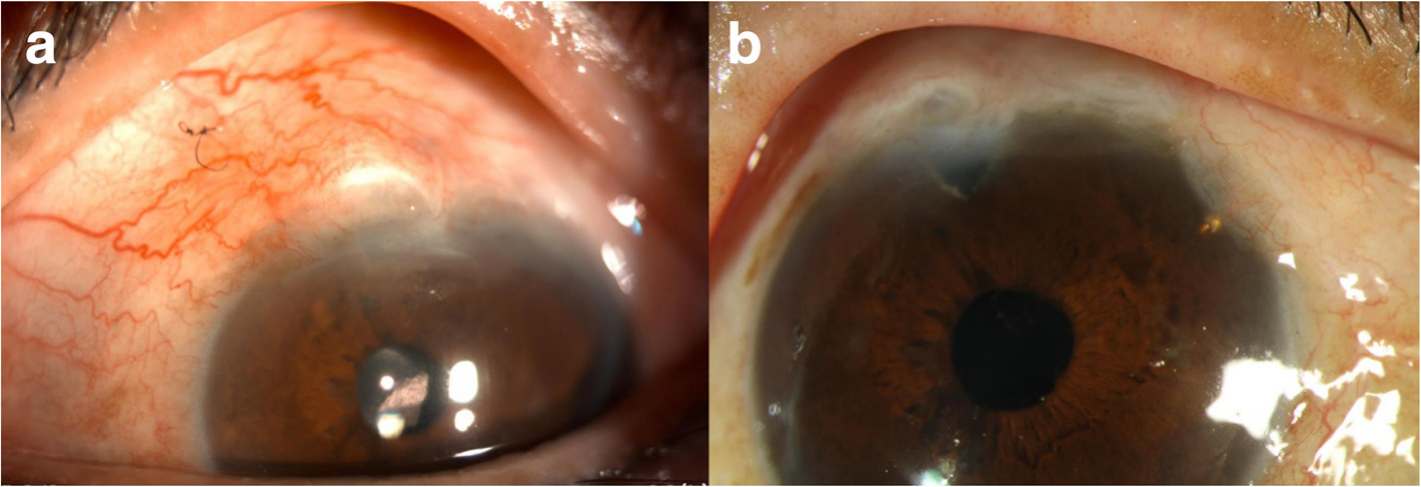
ExPRESS shunt placement. The IOP decreased from 45 to 17 mmHg after surgery. **a** Gradual increase in IOP after the first intervention with trabeculectomy. **b** The Express Shunt Mini Glaucoma shunt was placed at 2 o’clock, adjacent to the limbus

Due to the progressive course of glaucomatous change, we suggested that the patient first have filtering surgery for the left eye. We performed a trabeculectomy, which resulted in a postoperative IOP reduction (from 30 to 12 mmHg) on the first day. No complications occurred intraoperatively and none was reported at the one-month post-operative review. However, over the next three months, although the filtering bleb significantly elevated, the intraocular pressure (IOP) increased gradually, reaching approximately 40 mmHg (Fig. 2a). Subsequently, this patient received the placement of one ExPRESS miniature glaucoma device P200 (Fig. 2b), which resulted in the lowering of the IOP (from 45 to 17 mmHg), and no further deterioration of visual acuity occurred 6 months after this intervention, while the IOP of the other eye still ranged between 33 and 35 mmHg.

During the management of intractable secondary glaucoma, we reviewed the patient’s past medical history and image data and found that the patient was susceptible to vascular thrombosis and stenosis, as evidenced by the high venous pressure observed during haemodialysis, and while creating the vascular shunt, repeated angioplasty and thrombectomy for venous outlet thrombosis were required. There were multiple attempts at creating vascular accesses during the initial 2 years. First, a right neck Hickman catheter was inserted for temporary haemodialysis access, after which a second was placed in her right arm, but failed within a short time. Third, an arteriovenous fistula (AVF) was created in the left arm with a radial arterial-cephalic vein anastomosis, which failed in three years and was replaced by an arteriovenous graft (AVG) between the brachial artery and the axillary vein in the left arm after 6 months. The patient had been under haemodialysis via AVG since then, but encountered acute occlusion once and received a balloon percutaneous transluminal angioplasty (PTA). In 2011, the patient was admitted once for SVC syndrome after an unsuccessful attempt to place a Hickman catheter from the right and left internal jugular vein to bypass the complete obstruction of the right subclavian vein and right brachiocephalic vein. PTA was performed unsuccessfully, despite the spontaneous resolution of oedema on the left side of the face. However, the patient presented with intermittent recurrence and indolent face and neck oedema afterwards. Over the next 3 years, marked SVCS, accompanied by uncontrollable IOP, led to irreversible glaucomatous optic neuropathy. Although femoral double lumen creation and venous bypass construction were suggested as alternative haemodialysis access routes, along with the ligation of the previous AVF in the left arm, the patient refused any cardiovascular surgical options.

## Discussion

SVC syndrome due to the obstruction of blood flow occurs most frequently in intrathoracic malignancy, while SVC syndrome due to intravascular devices, such as in haemodialysis, is rare [4]. Although SVC syndrome is usually not life threatening and there is a lack of randomized clinical trials, Yu et al. previously proposed a classification for SVC syndrome; however, there is no consensus on the ocular manifestation and management [5]. Our case, which was graded as 2 according to Yu’s classification, demonstrated moderate haemodynamic symptoms, such as oedema of the head, including the eyelids and neck, without respiratory and neurologic signs. Although the timepoint of the onset of secondary glaucoma is unclear, we eventually observed irreversible visual deterioration.

As this patient would not accept suggestions for further interventions for the unmanageable venous occlusion, the IOP increase persisted, even with medication. Similar to results reported in a review for the management of elevated episcleral pressure [6], conventional antiglaucoma agents were not beneficial for our patient, who had unchanged SVC syndrome. Furthermore, after the first surgical intervention with trabeculectomy, the patient’s IOP gradually increased after 3 months. ExPRESS was performed secondarily, and the IOP was stabilized for 6 months, without postoperative complications.

Glaucoma filtration surgery has been a priority surgical intervention in primary glaucoma for decades. Trabeculectomy redirects the aqueous humour to the subconjunctival space to gradually form a bleb. However, this has a limited effect in patients with secondary glaucoma in SVC syndrome with venous drainage impairment. However, our results showed that after primary filtration surgery failed, the ExPRESS implant effectively facilitated aqueous outflow through the intrascleral space, thereby resulting in a normal IOP. In contrast, the IOP of the other eye, for which the patient refused any further intervention, remained high, despite treatment with medication.

## Conclusions

Secondary glaucoma with elevated episcleral pressure in SVC syndrome needs attention because the early ocular symptoms may be subtle. Although no randomized clinical trials for the treatment of elevated episcleral venous pressure exist, the ExPRESS miniature glaucoma implant may be an option for IOP control. Optimal management can thus prevent irreversible vision deterioration.

## Abbreviations

AVF, arteriovenous fistula; AVG, arteriovenous graft; BCVA, best-corrected visual acuity; CTA, computed tomography angiography; IOP, intraocular pressure; OD, right eye; OS, left eye; PTA, percutaneous transluminal angioplasty; RNFL, retinal nerve fibre layer; SVC, superior vena cava
